# Predicting drug shortages using pharmacy data and machine learning

**DOI:** 10.1007/s10729-022-09627-y

**Published:** 2023-03-13

**Authors:** Raman Pall, Yvan Gauthier, Sofia Auer, Walid Mowaswes

**Affiliations:** 1 Digital Technologies Research Centre, National Research Council of Canada, 1200 Montreal Rd, Ottawa, K1A 0R6 ON Canada; 2 PharmaGuide Inc, 55 West Beaver Creek Rd Unit 20, Richmond Hill, L4B 1K5 ON Canada

**Keywords:** Analytics, Drugs, Machine learning, Pharmacies, Shortages, Supply chain, Therapeutics

## Abstract

Drug shortages are a global and complex issue having negative impacts on patients, pharmacists, and the broader health care system. Using sales data from 22 Canadian pharmacies and historical drug shortage data, we built machine learning models predicting shortages for the majority of the drugs in the most-dispensed interchangeable groups in Canada. When breaking drug shortages into four classes (none, low, medium, high), we were able to correctly predict the shortage class with 69% accuracy and a kappa value of 0.44, one month in advance, without access to any inventory data from drug manufacturers and suppliers. We also predicted 59% of the shortages deemed to be most impactful (given the demand for the drugs and the potential lack of interchangeable options). The models consider many variables, including the average days of a drug supply per patient, the total days of a drug supply, previous shortages, and the hierarchy of drugs within different drug groups and therapeutic classes. Once in production, the models will allow pharmacists to optimize their orders and inventories, and ultimately reduce the impact of drug shortages on their patients and operations.

## Highlights

- Using sales data from 22 Canadian pharmacies and historical drug shortage data, we built supervised machine learning models able to predict shortages for the majority of the drugs that are in the most dispensed interchangeable groups in Canada.
- The modeling process (and related feature importance measurements) offer pharmacists and other health care practitioners a deeper understanding of the factors associated with drug shortages.
- Our models are designed to be implementable by pharmacies, which have access to demand-side data and reported shortage data, but generally do not have access to supply-side data (since they have limited visibility on the supply chain of drug manufacturers).
- Once deployed into production, the model will allow pharmacists to optimize their orders and inventories, and ultimately reduce the impact of drug shortages (a common and complex problem) on their patients and pharmacy operations.

## Introduction

### Background

Drug shortages are a complex, global problem [1, 2] with negative impacts on patients, pharmacists, and the entire health care system. Drug shortages can arise for many reasons, from delays in drug approval to issues with manufacturing, raw material supply, drug distribution, or front-line delivery by pharmacies [3, 4]. Unexpected high demands or demand fluctuations can also lead to drug shortages [1].

For patients, drug shortages can have major implications, for instance when drugs against severe allergies [5], severe pain [6, 7], mental illnesses [8], childhood diseases [9], or COVID-19 [10] become unavailable.

For pharmacists, drug shortages can present serious challenges as well. In Canada, when a drug with a particular Drug Identification Number (DIN) becomes unavailable, a pharmacist must find an alternative product within the same Interchangeable Group (IG) that comprises that DIN [11]. This does not require authorization from the patient’s physician, but a change in medication to a less-familiar alternative is generally not desirable and can have adverse effects in some scenarios. When all the drugs within an IG become unavailable, logistical and health challenges increase as the pharmacist has to explore alternative medications within a broader Therapeutic Class (TC) of drugs that may not be optimal for the patient’s condition. Such a change requires physician authorization and presents higher risks and health implications for the patient [12]. Such sourcing of alternative medication can occur multiple times a day [13] and the labor cost incurred can be significant (median of 3 hours per week per pharmacist [14]). Shortages also generate other negative impacts for the pharmacies in terms of safety risks, potential medication errors, and stress on health professionals [15].

Many countries have developed protocols that set expectations for drug manufacturers in anticipation of, or in response to, drug shortages. In Canada, such protocols were initially released in 2013 and updated in 2017 by Health Canada [16] to coincide with the launch of Drug Shortages Canada [17], a publicly accessible and centralized database for reporting drug shortages and discontinuations. Since March 2017, all Canadian drug shortages must, by law, be reported in this database [18]. Similarly, in the United States, the Food and Drug Administration (FDA) requires drug sellers to report in a centralized database when they are not able to meet demand for a product, or when they stop selling a product [19].

### Related work

The introduction of such public databases and mandatory reporting now offer a better means of understanding drug shortages. A retrospective study by Zhang et al. [12] identified factors associated with drug shortages and showed, for example, that drugs in the markets with branded manufacturers and a single generic manufacturer are significantly more likely to be in shortage than drugs in other market structures.

Beyond descriptive or retrospective analyses, there are many predictive applications of data analytics to the pharmaceutical supply chain. A review of the state-of-the-art by Nguyen et al. [20] shows that machine learning is barely starting to be leveraged by the pharmaceutical industry as a tool for shortage avoidance. It remains mainly used to improve the accuracy of demand forecasts [21–24] or to better segment products through clustering algorithms [25], and not to directly make shortage predictions.

### Contribution

With this work, our contribution is threefold. First, we offer a a deeper understanding of the factors associated with drug shortages by leveraging shortage data that drug manufacturers now have to publicly report, and by engineering new features from it. Second, we present a supervised learning approach that predicts the majority of impactful shortages for drugs that are in the most dispensed interchangeable groups. Third, we design this model to be implementable by pharmacies, which do have access to demand-side data and reported shortage data, but generally do not have access to supply-side data since they have limited visibility on the supply chain of drug manufacturers.

### Outline

Section 2 clarifies the prediction problem from the perspective of pharmacists. Section 3 describes the data preparation and validation. Section 4 focuses on feature engineering and the outcome variable. Section 5 describes how the predictive models are built and evaluated, and presents their most important features. Section 6 further discusses modelling performance and utility, as well as important considerations for model deployment into pharmacy systems.

## The prediction problem

Pharmacists are responsible for the front-line delivery of drugs to patients, and they must be able to manage drug shortages to ensure continuity of care. It is recommended that pharmacists monitor drug supply closely so they can act swiftly in the event of a shortage [26]. However, pharmacists do not have visibility on the supply chain of all their suppliers and they generally have no means of anticipating shortages before they are announced by drug manufacturers. Yet, obtaining early warning of drug shortages would be highly beneficial to pharmacists, as it would allow them to reserve certain drugs for their most vulnerable patients, especially those with chronic diseases and accustomed to specific medications, and find suitable alternatives for their other patients.

From the pharmacists’ perspective, and for the purpose of this work, we can summarize the prediction problem as follows: *predict the majority of impactful drug shortages early for the top-supplied drugs*, where:

- “majority” means the predictive model should ideally detect at least 50% of actual shortages;
- “drug shortage” means a situation in which the manufacturer to whom an official DIN was issued for the drug is unable to meet the demand for that drug [16];
- “impactful drug shortage” means a situation when at least half of the DINs within a particular IG become unavailable, often forcing pharmacists to consult physicians on using other drugs in a same TC, but not directly interchangeable with the DIN of interest;
- “early” means detecting shortages 30 days or more prior to actual shortage; and
- “top-supplied drugs” means drugs that are in the most dispensed IGs, in terms of their days of supply.

To solve this prediction problem, pharmacists generally have access to demand-side data only, that is, data related to drug sales and prescriptions. They also have access to historical shortage data from public databases such as Drug Shortages Canada [17] or its FDA equivalent [19].

Forecasts need to be made sufficiently in advance to inform pharmacies’ inventory management and drug ordering processes. Drug inventory management is a complex process requiring metrics associated with dispensed amounts, stock levels, shelf time, budgets, and other factors [27] that are typically updated on a monthly basis. Moreover, when there is a disruption in drug supply, pharmacists are asked to limit the supply of medications to 30 days [26], which generally suffices to meet patients’ immediate needs while avoiding unnecessary stockpiling. A forecasting horizon of 30 days also offers pharmacists enough early warning to elaborate continuity of care strategies with doctors, patients, suppliers, and other stakeholders.

Drug manufacturers and suppliers face a similar prediction problem. They do have access to a broader set of supply-side data (e.g., manufacturing capacity, raw material availability), but mainly data pertaining to their own drugs. Accordingly, although this work aims to build predictive models for the purposes of pharmacies, such models can still be useful to drug manufacturers and suppliers, as they may allow them to anticipate shortage situations from competitors, and possibly adjust their production and distribution of interchangeable drugs.

## Data

All of the data used for this work, with the exception of shortage data, were obtained from PharmaGuide [28], a Canadian company that provides software solutions for the delivery of pharmacy services. We had access to anonymized data covering 22 pharmacies across Ontario. This includes a mix of pharmacies of various sizes and settings (both community and institutional). The data covered years from 2014 to 2021, inclusively.

**Fig. 1:**
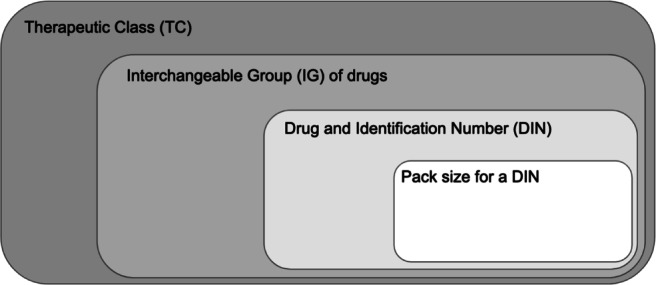
Hierarchy of drugs and therapeutics. Shortages are typically reported at the lowest level

Shortage data was downloaded from the Drug Shortages Canada [17] website. The data covers 12,269 drug shortages reported to Health Canada between 2013 and 2021, with their corresponding start dates and end dates. For the purpose of this work, only actual and resolved shortages are relevant (anticipated and avoided shortages were removed from the dataset prior to modelling). All shortages in the DSC database are reported on a DIN/pack-size basis, i.e., each record in the database pertains to a drug in specified pack sizes. Figure 1 illustrates how this reported data fits into the hierarchy of drugs and therapeutics.

Table 1 lists the main predictor variables, as well as any new features engineered from the data. It includes three sub-datasets. The *Drugs* dataset brings together all of the unique DINs for all available drugs, the corresponding drug classification information used by the pharmacies, as well as the strength of the DIN and its common name. The *Monthly Dispensed Data* combines all of the dispensed drug information from pharmacy data, such as days of supply, number of patients, prescription (Rx) counts and quantity dispensed, for each unique DIN and IG code, aggregated by month and year available for that particular DIN. Lastly, *Top DINs and Shortages* refer to an ordered list of DINs (and corresponding IG codes). The DINs in this list represent the most-supplied drugs by the 22 pharmacies considered, calculated by summing the days of supply for each IG, ordering by the largest sum, and indicators of shortages and their timeframes. Of note, the fields in the *Top DINs and Shortages* are stratified by pack size, whereas this is not the case for the other two datasets.

**Table 1:** Three datasets created prior to the modelling stage, along with field descriptions

Dataset	Fields	Description
*Drugs*	din	drug identification number (DIN)
	group code	interchangeable group (IG) code for a DIN
	therapeutic class	therapeutic class code from the American Hospital Formulary Service (AFHS) system [29]
	strength	strength of the drug
	drug code	drug code classification system
	tc atc number	therapeutic code from Anatomical Therapeutic Chemical (ATC) system [30]
	common name	common name for the drug/DIN
*Monthly*	month year	month and year of dispensed DIN
*Dispensed*	sales month	month of dispensed DIN
*Data*	sales year	year of dispensed DIN
	day supply	days of supply of dispensed DIN
	num of patients	number of patients to which DIN was dispensed
	quantity dispense	quantity of dispensed DIN
	rx count	count of dispensed DIN
	din	drug identification number (DIN)
	group code	interchangeable group code per DIN
	fulldate*	full date of when drug/DIN was dispensed
	tot dos*	days of supply (DOS) for each DIN and each month
	tot patients*	number of patients for each DIN and each month
	avgdos pat*	average days of supply per patient, for each DIN and each month
	tot qty disp*	quantity dispensed for each din and each month
	tot rx*	prescription (Rx) count for each din and month
*Top DINs*	din	drug identification number (DIN)
*and*	pack size	package size
*Shortages*	group code	interchangeable group code per DIN
	group demand*	days of supply dispensed for each group code / IG
	demand*	days of supply dispensed for each DIN
	shortage*	flag indicating if DIN was in shortage before
	start date*	actual shortage start date from DSC database; if missing, then use anticipated shortage start date; if missing then use report date of shortage
	end date*	actual shortage end date from DSC database; if missing, use updated date of report shortage
	total shortage*	number of days of shortage, as a function of start and end date

Features engineered from raw data are denoted by an asterisk

In this work, only the top 100 IGs were considered; we refer to these 100 unique drug groups throughout this paper as the “IGs under consideration”. These IGs correspond to 784 component DINs, which we refer to as the “DINs under consideration”. The final dataset required for modelling (with the exclusion of the outcome variable, which will be discussed later) was obtained by joining the *Drugs*, *Monthly Dispensed Data*, and *Top DINs and Shortages* datasets to include only the DINs under consideration.

### Data limitations

There are two main data limitations, both related to historical shortage data. The first has to do with completeness, since the mandatory reporting drug shortages began in March of 2017 [18]. Shortage data prior to this date may not be complete, timely, or accurate. We kept that data for exploration purposes only. For predictive modelling, we used data reported since March 2017. We assume that manufacturers complied with the mandatory reporting protocol and reported all actual shortages for the drugs we considered.

The second limitation of the DSC data concerns data quality: there were nearly 400 shortages for which the start date came after the reported end date. When filtering on the DINs under consideration, only one record had this problem, which was removed from the analysis.

### Shortage data validation and pre-processing

To minimize DSC data quality issues, we performed additional pre-processing. For example, to validate the “actual” shortage start dates and end dates (or impute any missing dates), we leveraged other temporal information available in other DSC records, such as the “anticipated start” date, as well as corresponding anticipated end dates; and dates at which the shortage event was initially entered in the database, and last updated. In instances where the actual end date was unavailable for a given shortage, we used the last updated date as the end date of the shortage. For start dates we used the actual start date; if missing, the anticipated start; and, if also missing, the date of entry. There was a few records where the start dates and end dates remain missing or intractable (where the start date is after the end date) – specifically, for 19 of all shortage records in the DSC. The majority of these are related to anticipated shortages as opposed to actual shortages, and only one of these records pertains to a DIN under consideration. These records were removed from the dataset.

Additional pre-processing was needed because shortages are not reported by DINs, but in terms of particular DIN formats, or pack sizes. Pack sizes are entered as free-form text, and can take various forms for the same item. For example, DIN 15237 (Aventyl 25 mg) has had four shortages (all in 2018); in the first two, the shortage is of package “100 Bottle”, whereas in the second, there is a shortage of package “100 BTL”. As pharmacists are mainly concerned with the overall availability of this DIN, we can match these packages to the listing of the DINs in the catalog of McKesson [31], a major drug distributor, where package sizes are listed as numbers (continuing our example above for DIN 15237, there are three pack sizes available for this DIN: two of size 100 by different manufacturers, and one of size 500). In order to match these quantities together – the free-form pack sizes in the DSC data, and the numeric catalog numbers – parsing of the free-form text in the DSC data was required, converting them to their numeric components. In most cases, we were able to automatically match the shortage of a DIN to one or more specific pack sizes in the catalog; however, there were 46 of the 749 shortage entries in the DSC data which pertain to DINs under consideration (or approximately 6% of all shortages for these DINs) where the pack size stated were not listed in the McKesson catalog. In these ambiguous instances, we assumed that the DIN was in shortage in all of its packaging sizes.

## Identifying shortages and their features

### Shortage impact score

The shortages reported in Drug Shortages Canada [17] are associated to specific DIN/pack-size combinations, for specified time intervals. A “shortage” is a situation where the manufacturer is unable to meet the demand for a DIN/pack-size for the specified period, during which we assume the DIN/pack-size will be *unavailable* to pharmacists placing orders (a portion of the demand may still be partly and temporarily filled at pharmacies still having stocks).

For modelling, we need a measure of shortage impact over time, for each DIN and for each interchangeable group (IG). This requires assessing the contribution of each specific pack size of a DIN to its overall IG. This is based on two factors: the contribution of each pack size to overall orders by the pharmacies, and the frequency at which the DIN within that IG has been historically used by patients.

The first of these factors was not in the data, thus we assumed that all different pack sizes of a given DIN are equally important to the pharmacies (e.g., if DIN *x* can be purchased in bottles of 100 or 300, we assume them to be equally important, and having a shortage in one or the other would yield a total shortage impact score of 0.5 for the DIN). The second factor is easier to compute and is based on the total DOS for the DINs in each IG. For instance, if an IG *w* is made up of DINs *x*, *y*, and *z*, and DIN *x* makes up 40% of past DOS for the IG, then we say that this factor for DIN *x* is 0.4. The total strength of a shortage of an IG is then taken to be the sum of all products of these factors for DINs/packages that are in shortage at any given time. Continuing our previous example, if DIN *x* for IG *w* is in shortage in pack size 300 but is available in pack size 100, and DINs *y* and *z* are fully available, then on that date the shortage indicator of IG *w* would be 0.5 × 0.4 = 0.2.

**Fig. 2:**
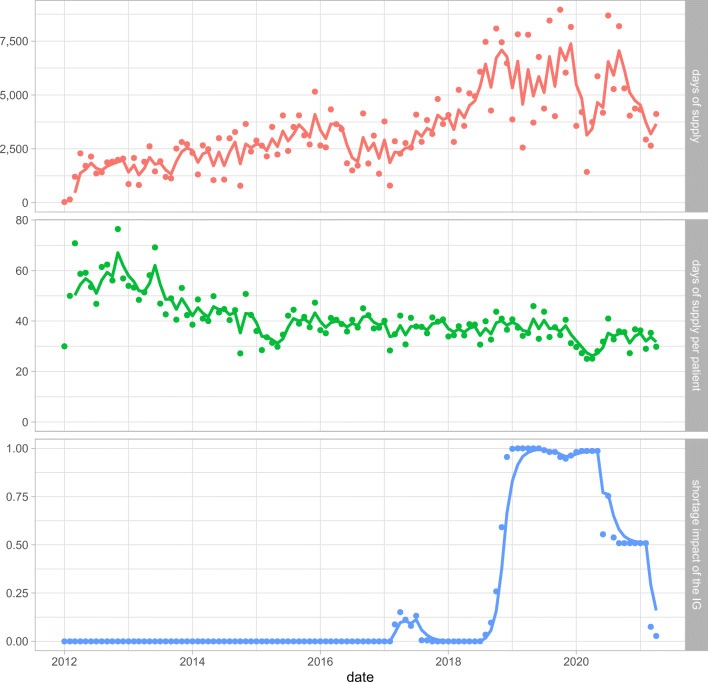
Several measures of interest for IG 344: *Angiotensin II Receptor Telmisartan* – days of supply, days of supply per patient, and the computed shortage impact score for the IG. The dots represent the monthly quantities for each measure, while the solid line represents the three-month exponential moving average

This yields a time series with monthly resolution for each IG, with values ranging from zero (all of its component DINs are available in all pack sizes) to one (nothing in the IG is available) that can and will be used as an outcome variable for modelling. An illustration of this shortage impact score for one specific IG over time is shown in Fig. 2. It includes a composite view of the days of supply (DOS), DOS per patient, and shortage impact score for IG 344 (Telmisartan). Note that the shortage impact score (in blue) increases slightly in early 2017 (denoting a shortage in some pack sizes), rises to a complete shortage in 2019, before dropping again to a partial shortage that is resolved in early 2021. The total days of supply dispensed (in red) increased before and during the shortage, while the days of supply dispensed per patient (in green) remained relatively stable before dropping during the large shortage.

Note also that the shortage impact score is an aggregated outcome variable applying to the entire set of pharmacies, for a specific IG over time. The underlying assumption is that when a shortage is reported in the DSC database, all pharmacies become equally unable to order the drug.

### Features of interest

#### DIN-level features

The first set of features that must be considered for modelling pertains to DINs. Many DIN-specific features can be directly obtained from pharmacies’ data, including the days of supply, number of prescriptions, number of patients, average days of supply per patient, and quantity of drugs dispensed, or their respective changes from month to month.

**Fig. 3:**
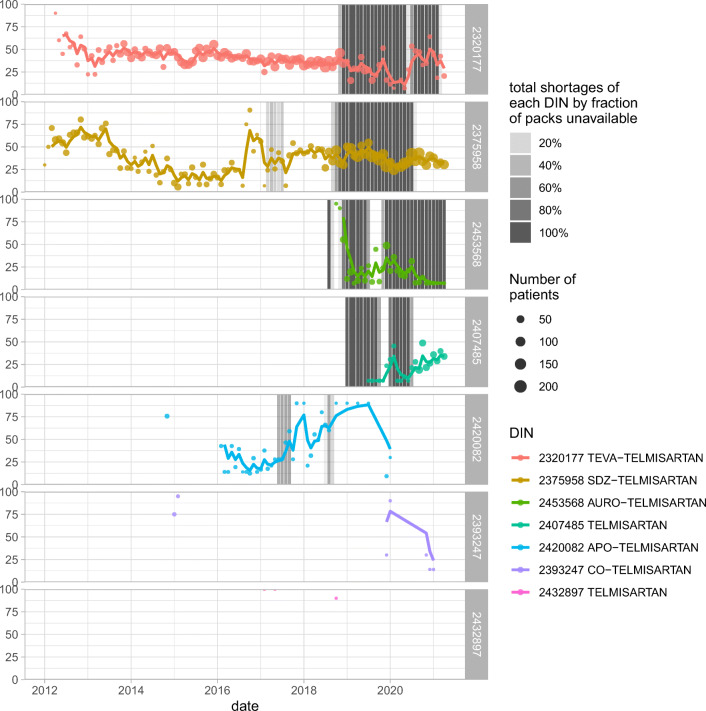
Mean monthly days of supply (DOS) provided per patient for all DINs in IG 344, overlaid with the strength of the shortages seen in the DINs in each month. Each month’s data are displayed as circles, with three-month exponential moving averages displayed as solid lines. The DINs are ordered in descending sequence by their total DOS

For example, Fig. 3 shows the number of patients associated to a selected set of DINs over time, and the DOS per patient, overlaid by the shortages seen for each of the DINs (i.e., the fraction of pack sizes which are unavailable for that DIN at that moment). The dates at which shortages occur seem to be associated with large scale changes in the number of patients taking specific DINs.

#### IG-level features

The same features mentioned for DINs can be used in aggregate across each IG. The total amounts dispensed by each pharmacy for an IG is easily computed as the sum of the total DOS of each component DIN’s DOS. The same is true for the number of patients taking a medication from a given IG in each month, as well as the DOS per patient ratio.

#### Month-to-month changes in the ratio of the DINs

Another feature that may be indicative of drug shortage is the month-to-month variation in the ratio of DINs being dispensed within an IG over time. We developed a measure that specifies the maximum change in the percentage of patients being given that DIN across all DINs in the IG, for each IG in each month. As an example, if IG *w* had three DINs (*x*, *y*, and *z*) which were given to 80*%*, 10*%*, and 10*%* of patients in month *t*, and 50*%*, 30*%*, and 20*%* of patients in month *t* + 1, then the maximum month-to-month change in the ratio of the DINs is ${\max \limits } (| 80-50 |, | 10-30 |, | 10-30 |) = 30\%$ (the maximum change was for DIN *x*).

#### Strength of preference of DINs

A related feature is the strength of preference of the DINs, which is a way to measure a pharmacist’s preference for one DIN over all others in the IG. One way to compute this is via the *Gini coefficient* [32], a measure of statistical dispersion commonly used in economics. In our case, we can use it to measure disparity in the amount of patients associated to each DIN.

In periods of certainty (with no shortages), we postulate that pharmacists have a preference for one DIN over others in the IG (either due to relationships with the supplier, habit, patient preference, or other); whereas in periods of shortage patients may have to be given whatever DINs remain available in the IG, regardless of preference. Computing the Gini coefficient *G*_*i*_ for the *i* th IG in each month is a way to measure the strength of DIN preference within the IG. In this approach, *G*_*i*_ = 1 represents a total preference of one DIN over all others for the IG; whereas *G*_*i*_ = 0 means that there is a uniform (equal) assignment of DINs in the IG to the patients.

**Fig. 4:**
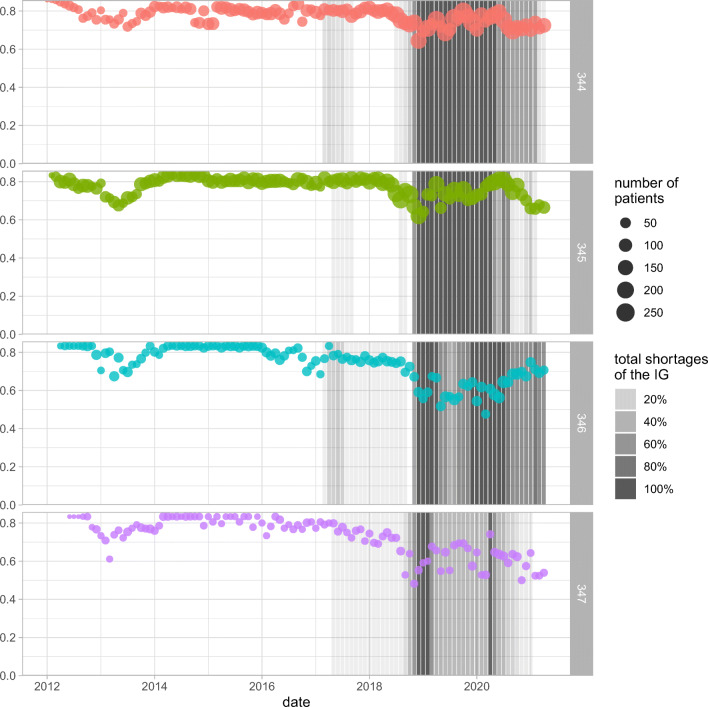
Statistical dispersion of ratios of the DINs computed using the Gini coefficient for four separate but related IGs (344, 345, 346, and 347), overlaid by the shortage impact scores for the IGs in each month

We provide in Fig. 4 the Gini coefficient in each month for four separate but related IGs (344, 345, 346, and 347; each of which is a Telmisartan drug in different dosages). These values are overlaid by the shortage impact scores for the IGs in each month. It is evident in the figure that periods of low Gini coefficient are co-incident with shortages after March 2017 (when shortage reporting became mandatory for suppliers), and that there is a significant drop in the Gini coefficient immediately before periods when shortages occur.

#### Shortages in neighbouring IGs

When drugs are unavailable for a given IG, pharmacists may turn to neighbouring IGs in the same Therapeutic Class (TC) of the AFHS system [29]. Drugs in neighbouring IGs can be used to treat the same conditions, but may differ in the specific molecule, chemical mechanism, or dosage amounts. Hence, shortages in one IG may influence the future appearance of shortages in neighbouring IGs, and so may have predictive power in our statistical models. Specifically, shortages in neighbours at different “distances”, i.e., at different levels of aggregation (TC, TC up one level, TC up two levels, and TC up three levels), are considered. For example, for TC 84:04.08.28, TCs one level up start with 84:04.08.**; TCs two levels up start with 84:04.**.**; and TCs three levels up start with 84:**.**.**; where the “*”s represent any number.

#### Seasonality in the DOS or in the patients

There are seasonal patterns in the dispensed amounts for many types of drugs (e.g., anti-depressants, anti-allergies, anti-anxiety). Considering the prevalence and impact of these seasonal effects may increase the fidelity of predictive modelling, so we include changes in DOS from one month to the next.

### Lagging the features

When developing predictive models for times series, the features for multiple previous months can be leveraged to predict an outcome. This is called *lagging* the features [33] and in the present work we lagged all features in our models by up to four months, meaning that all values for the series up to four months past were included as features in the model, in effect quadrupling the number of features considered.

## Modelling

For modelling, we considered a total of 100 IGs (and 784 corresponding DINs) to include a breadth of drugs and drug shortages (and sizes thereof).

### Training, testing and holdout sets

Data prior to 2017 was not used for modelling, since reporting of drug shortages was not made mandatory until then. The data from 2017 to 2020 was used for iterative training and testing. The holdout set included four months of data, encompassing the period from January to April 2021. The outcome variable is the shortage impact score described in Section 4.1.

**Fig. 5:**
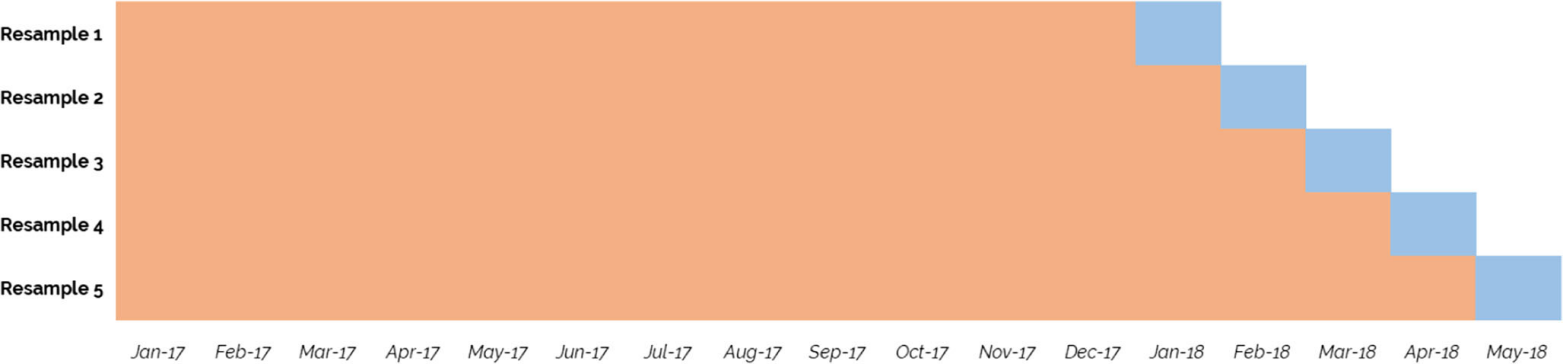
Example of a time-series blocked cross validation scheme. Salmon-coloured areas in the figure represent the training periods. Blue areas represent the testing periods

We constructed paired train-test sets using a time-series blocked cross validation technique [33]. Figure 5 shows an example of a validation configuration for a set of five resamples. In our case, to include the entire period of January 2017 to December 2020, we constructed 36 resamples on which the models were trained. We did not stratify training data by pharmacies, partly because the shortage impact score is an aggregate measure across all pharmacies, and because it would have been difficult to break the set of pharmacies into clearly-defined classes with non-overlapping attributes.

Having various paired train-test sets allows us to build models that incorporate information stemming from different months of observations, with each resample including one more month of training data than the last, without focusing on the particularities in very specific circumstances which may have led to changes in the modelled outcome. This allows for better generalizability and assessment of the models. The models are therefore trained and tested several times, and the model accuracy metrics are averaged over the different resamples in order to provide overall measures of performance. The test set is independent of the training set for each resample.

The final holdout set (four months) was used only once at the end to assess “real-world” model performance once deployed and its generalizability. We will refer to the outcome in the holdout set as *forecasts* in the rest of this paper.

### Modelling engine

All models were built using the R statistical computing environment [34], mainly in the tidymodels modelling framework [35]. Several techniques exist for time series forecasting and regression modelling [36, 37]. We experimented with various algorithms, such as classic time series approaches (ARIMA and error trend seasonal (ETS) time series models [33]) as well as general statistical learning approaches [38–40]. We ultimately selected *eXtreme Gradient Boosted trees* (XGBoost) [41] as the model’s engine [42], based on its initial performance on the IGs previously described. XGBoost is an advanced regression technique which has gained popularity due to its simplicity in implementation, its accuracy, speed, and scalability [41]. It produces a prediction model in the form of an ensemble of weak prediction models, typically decision trees, and uses boosting to create a strong model from the weak models. By adding models on top of each other iteratively, the errors of previous models are corrected by the next model, until the training data is accurately predicted or reproduced by the model. XGBoost is one of the most prevalent types of models in use for both regression and classification problems today (e.g., [43, 44]). Based on decision trees, XGBoost can also handle both numerical and categorical data and is robust against co-linearity [38, 39]. It also has the advantage of being supported in most cloud environments when models are deployed.

### Modelling results and performance

Predictive models for the shortage impact score were built for each IGs taking into account the features presented in Section 4. Recall that several of these features are IG-specific, whereas others concern the specific DINs of the IGs. As the number of DINs comprising the IGs may differ, the number of features present in each IG’s models differs accordingly.

**Fig. 6:**
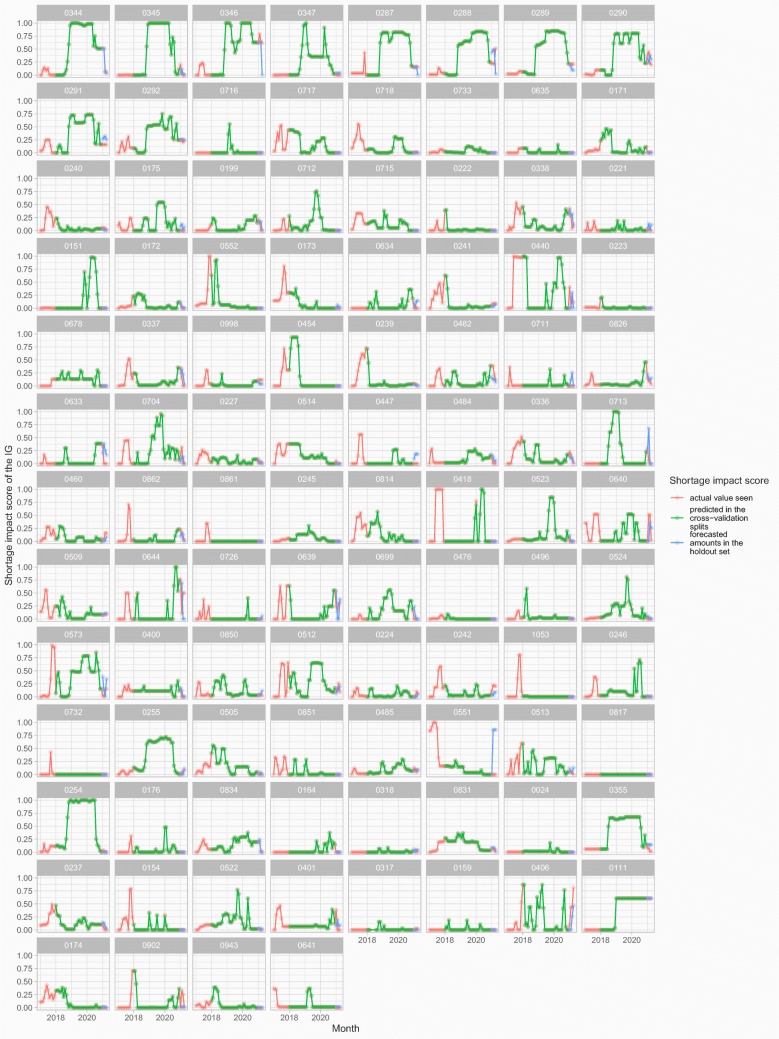
Modelling results for 100 IGs showing the actual shortage impact scores seen (in red), the model’s predicted scores in the train/test cross-validation splits (in green), and the forecasted scores for the holdout set (in blue)

We present all shortage impact scores (for each of the 100 IGs) observed over the entire training and testing span, as well as the forecasts for the holdout set calculated by each of the IG’s predictive models in Fig. 6. The red points represent actual shortage impact scores, while the green points are predictions from the model in the train/test cross-validation splits. The blue points are the forecasted shortage impact scores for the holdout set. Note that the forecasts overlap the actuals in many instances, but differ at times (see IG 0346 in the top row for example).

Several measures exist to assess the performance of predictive models. These measures generally seek to measure error in predictions in absolute, scaled, or relative terms [45]. We selected the *Mean Absolute Error (MAE)* as our performance indicator to measure forecasting accuracy, due to its simplicity and intuitiveness [46]. We summarize the MAE over the entire holdout period to get an average accuracy metric.

**Fig. 7:**
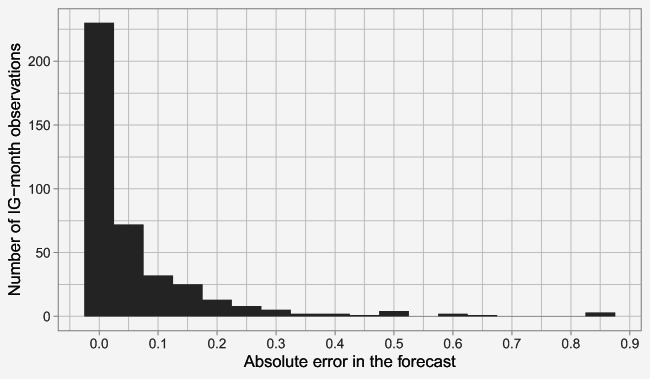
A histogram showing the distribution of MAEs in the forecasts, across all IGs and months in the holdout set

We provide an illustration of the model performance in Fig. 7, which depicts the distribution of the MAE over all IGs, in all forecasted months (i.e., over 100 × 4 = 400 forecasts). As seen in the figure, approximately 80% of forecasts have average shortage impact score predictions that are off by less than 0.10; the other 20% of forecasts have larger errors. A more detailed discussion of the forecasting errors will be provided in Section 6.1.

### Feature importance

The *importance* of each variable is measured by how much the model’s performance decreases when the variable is not available, using the approach of Greenwell [47]. This was done iteratively for all boosted trees across all 100 IGs. Although each IG has its own model and its own variables of importance, in aggregate there are many similarities across the IGs. We present in Table 2 the average importance of the features across all 100 IGs, in descending order.

**Table 2:** Importance of the variables seen in the models, averaged across all 100 IGs

Variable		
*Name*	*Description*	Importance
Shortage_impact	the IG’s shortage impact score	39.6*%*
Avgdos_pat.DIN	average days of supply per patient, for component DINs	13.8*%*
Shortage_tc1	shortages of neighbouring IGs, one level up in the TC hierarchy	9.7*%*
Tot_dos.DIN	total days of supply for component DINs	6.8*%*
Shortage_tc3	shortages of neighbouring IGs, three levels up in the TC hierarchy	5.6*%*
Shortage_tc2	shortages of neighbouring IGs, two levels up in the TC hierarchy	4.6*%*
Tot_qty_disp.DIN	total quantities dispensed of component DINs	4.2*%*
Chg_dist_dos	month-to-month changes in the ratio of the DINs, by DOS	2.9*%*
Shortage_tc	shortages of neighbouring IGs, in the same TC	2.6*%*
tot_rx.DIN	total number of prescriptions of component DINs	2.5*%*
Gini_pat	Gini coefficient (strength of preference of DINs) by number of patients	2.1*%*
Chg_dist_pat	month-to-month changes in the ratio of the DINs, by number of patients	1.9*%*
Gini_dos	Gini coefficient (strength of preference of DINs) by days of supply	1.7*%*
Dos_per_pat	days of supply per patient	1.1*%*
Qty	quantity dispensed for the IG	0.4*%*
Dos	days of supply for the IG	0.4*%*
Rx	number of prescriptions for the IG	0.1*%*
*Total*		100*%*

The greatest contribution to the model’s performance are the shortages seen in the IG’s previous months (39.6%), which is not surprising as shortages often last multiple months before being resolved. Thus a shortage in one month is a good indication of a shortage in the subsequent month. Shortages of neighbouring IGs also make important contributions to the models’ performance, with the closer ones in the TC hierarchy having generally greater importance: shortages in neighbouring IGs account for approximately one-fifth of the performance of the models.

Also of importance are variables concerning the days of supply for the IG’s component DINs: the average days of supply per patient (13.8%), and the total days of supply (6.8%). As was seen in Fig. 2, changes in the days of supply often precede shortages and, together, these variables concerning the days of supply per patient account for another one-fifth of the performance of the models.

Other features, notably month-to-month changes in the ratio of the DINs and the strength of preference of the DINS, had generally less importance, accounting for the final approximate one-fifth of the performance.

The importance of the variables can be separated by lagged month, as shown in Table 3. Not surprisingly, the most recent (*Lag 1*) variables are of most importance to the overall prediction, responsible for almost three-quarters of the performance of the models, with diminishing returns for variables with (*Lags 2*) to (*Lags 4*).

**Table 3:** Importance of the variables seen in the models, separated by lagged month, and averaged across all 100 IGs

Variable		Importance by Lag			
*Name*	*Description*	*Lag 1*	*Lag 2*	*Lag 3*	*Lag 4*
Shortage impact	the IG’s shortage impact score	38.1*%*	0.7*%*	0.3*%*	0.4*%*
Avgdos_pat.DIN	average days of supply per patient, for component DINs	6.4*%*	2.6*%*	2.0*%*	2.8*%*
Shortage_tc1	shortages of neighbouring IGs, one level up in the TC hierarchy	9.0*%*	0.6*%*	0.1*%*	0.0*%*
Tot_dos.DIN	total days of supply for component DINs	3.4*%*	1.2*%*	0.6*%*	1.5*%*
Shortage_tc3	shortages of neighbouring IGs, three levels up in the TC hierarchy	3.7*%*	0.8*%*	0.0*%*	1.0*%*
Shortage_tc2	shortages of neighbouring IGs, two levels up in the TC hierarchy	4.0*%*	0.3*%*	0.2*%*	0.1*%*
Tot_qty_disp.DIN	total quantities dispensed of component DINs	1.5*%*	1.3*%*	0.4*%*	1.0*%*
Chg_dist_dos	month-to-month changes in the ratio of the DINs, by DOS	1.2*%*	0.8*%*	0.3*%*	0.7*%*
Shortage_tc	shortages of neighbouring IGs, in the same TC	2.5*%*	0.0*%*	0.1*%*	0.0*%*
Tot_rx.DIN	total number of prescriptions of component DINs	1.2*%*	0.7*%*	0.3*%*	0.3*%*
Gini_pat	Gini coefficient (strength of preference of DINs) by number of patients	0.7*%*	0.8*%*	0.5*%*	0.1*%*
Chg_dist_pat	month-to-month changes in the ratio of the DINs, by number of patients	0.7*%*	0.4*%*	0.6*%*	0.2*%*
Gini_dos	Gini coefficient (strength of preference of DINs) by days of supply	0.5*%*	0.4*%*	0.5*%*	0.3*%*
Dos_per_pat	days of supply per patient	0.3*%*	0.4*%*	0.3*%*	0.1*%*
Qty	quantity dispensed for the IG	0.3*%*	0.1*%*	0.1*%*	0.0*%*
Dos	days of supply for the IG	0.2*%*	0.1*%*	0.1*%*	0.0*%*
Rx	number of prescriptions for the IG	0.1*%*	0.1*%*	0.0*%*	0.0*%*
*Subtotals*		73.7*%*	11.3*%*	6.2*%*	8.8*%*
*Total*		100*%*			

## Discussion

### Comparing forecasts to actual values

To better analyze forecasts, we categorize impact scores into an ordinal set of bins specifying the size of the shortages encountered, which we arbitrarily defined as:

- **None** when the shortage score was 0;
- **Low** when the shortage score was in the (0,33] range;
- **Medium** when the shortage score was in the (0.33,0.67] range; and
- **Large** when the shortage score was in the (0.67,1] range.

**Fig. 8:**
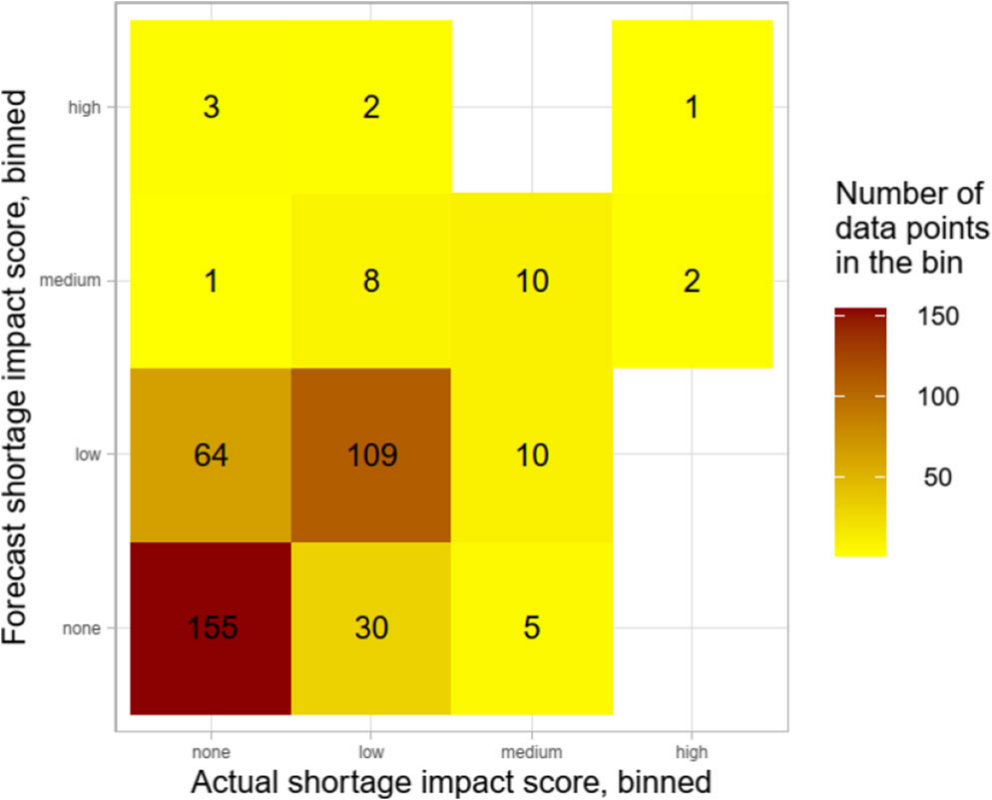
A confusion matrix of actual and forecasted binned values for each IG in each holdout set month (400 predictions)

Figure 8 shows the frequency at which we observe paired forecast-actual binned values for each of the 100 IGs under consideration in each of the 4 holdout set months (for a total of 400 predictions). We can see that high shortages are sometimes predicted as medium shortages, and medium shortages are sometimes predicted as either low or non-shortages. Conversely, we see that the model frequently and correctly predicts non-shortages as being non-shortages, but also frequently and incorrectly predicts them as being low shortages.

The overall accuracy of the model based on the matrix of Fig. 8 is 0.688 with a 95% confidence interval of (0.640, 0.733) and Cohen’s kappa value is 0.443. Class-specific metrics are presented in Table 4. The precision and recall of the model diminishes as the shortage impact increases, but specificity increases. This is a good thing from a pharmacist perspective, as a model frequently generating false alarms and incorrectly predicting high-impact shortages could not be relied upon.

**Table 4:** Class-specific metrics for different shortage impact levels

Bin (Actual)	*Precision*	*Recall*	*Specificity*
None	0.816	0.695	0.802
Low	0.596	0.732	0.705
Medium	0.476	0.400	0.971
High	0.167	0.333	0.987

Table 5 focuses on the model errors in each bin. The high (shortage impact score) bin has an average predicted shortage impact score of 0.597 with a corresponding MAE of 0.191. Thus, if an IG has an actual shortage impact score in the highest bin (on average, the shortage impact score is 0.788), we predict that 60% of DINS/packs will be unavailable, and the average error for this prediction is 19%. Simply put, if on average 79% of DINs/packs are unavailable, we in fact predict anywhere from an average of 41% to 79% of them as being unavailable.

**Table 5:** Model errors by binned actual shortage, along with the mean shortage score actually seen for the bin, and mean shortage score forecasted for the bin

	Mean shortage score		
Bin (Actual)	*Actual*	*Forecast*	MAE
None	0.001	0.037	0.037
Low	0.109	0.119	0.080
Medium	0.489	0.285	0.205
High	0.788	0.597	0.191

### Predicting the most impactful shortages

We return to our originally stated goal at the outset of this paper, which is “to predict the majority of impactful drug shortages early for the drugs in the most dispensed IGs.” We characterized a shortage as “impactful” if at least half of the drug’s IG is in shortage; i.e., when the IG has a shortage impact score that is at least 0.5. Out of the 400 IG × month instances in the holdout set, there are 17 instances with an impact score greater than or equal to 0.5. These are displayed in Table 6, along with their forecasted scores.

**Table 6:** Results for the actual “impactful shortages”

			Shortage Score		“Impactful Shortage” Predicted Correctly
IG Name and Details		Month	*Actual*	*Forecasted*	
0344	TELMISARTAN 40MG	Jan 2021	0.509	0.510	
		Feb 2021	0.509	0.510	
0346	TELMISARTAN/HCTZ 80MG/12.5MG	Jan 2021	0.617	0.620	
		Feb 2021	0.793	0.631	
		Mar 2021	0.647	0.637	
		Apr 2021	0.613	0.006	
0288	IRBESARTAN 150MG	Apr 2021	0.515	0.021	
0640	INDAPAMIDE 1.25MG	Feb 2021	0.510	0.011	
		Mar 2021	0.510	0.364	
0644	SPIRONOLACTONE 25MG	Jan 2021	0.758	0.691	
		Mar 2021	0.500	0.000	
		Apr 2021	0.500	0.002	
0406	NAPROXEN 500MG	Apr 2021	0.812	0.469	
0111	METHOTREXATE SODIUM 2.5MG	Jan 2021	0.606	0.606	
		Feb 2021	0.606	0.606	
		Mar 2021	0.606	0.606	
		Apr 2021	0.606	0.606	

We note that 10 out of 17 instances, or 59%, were correctly forecasted as being impactful shortages. Thus, we met the originally stated goal of early detection (30 days prior to actual shortage) and prediction of actual (impactful shortages), for 50% of the top-supplied drugs (that is, 100 top-supplied IGs categorized as having an impactful shortages). Recall that this is done without any visibility on the pharmacies’ supply chain and predictions are one month in advance. Given additional data around the inventories of suppliers, or a shorter forecast horizon, the predictive performance of the model would increase.

### Deployment considerations

How such predictive models should be deployed into pharmacy software is outside of the scope of this paper. Nevertheless, there are important considerations about how the results of predictive modelling should be leveraged and interpreted by end users.

Specifically, simply computing the impact shortage score and presenting it to pharmacists without clear and sufficient context would be risky, for two main reasons. First, the score could be interpreted as a probability of completely running out of drugs in an IG within the next 30 days, but the predicted impact score actually differs from a true probability of shortage. Second, if the predicted score is close to 1.0, it could be interpreted by certain users as an alert to stockpile drugs belonging to that IG. Multiple pharmacists making the same interpretation could, hypothetically, place several orders and increase demand enough to create a drug shortage that would not have occurred otherwise. In other words, the shortage prediction could become a self-fulfilling prophecy and artificially create undesirable situations.

To avoid such a scenario, converting the score to a qualitative scale as we did before is desirable (e.g., a four-point scale: “no shortage risk”, “low risk”, “medium risk”, “high risk”). With that approach, the highest rating on the scale should be used with parsimony. If given too frequently or inaccurately, it could create alert fatigue and ultimately be ignored, defeating its purpose. Note that based on the results previously shown, a “high” score was obtained in less than 2% of the forecasts.

Integrating shortage scores into the systems used for drug ordering and inventory management would make sense, as the main decision that can be informed by the model is how much of a drug a pharmacy should order. This decision needs to take into account the supply-side of the pharmacy environment (risk of shortage, existing inventories), but also the demand side. We are working at better predicting the demand for drugs at each pharmacy using machine learning [48]. These demand forecasts, combined with the risk of shortage for certain drugs and other factors (e.g., inventory levels, pharmacist’ risk tolerance) will be key in informing the drug ordering process.

Finding the right approach to presenting shortage scores and integrating them into existing systems will likely require experimentation. Slowly rolling out this feature to a small set of pharmacists, with iterative feedback and improvements, will increase the chances of its success and uptake.

## Conclusion

Drug shortages and related supply chain issues can have significant negative impacts on patients, pharmacists, and the broader health care system. Using sales data from 22 Canadian pharmacies and historical drug shortage data reported to Health Canada, we showed that machine learning models can help pharmacies to predict shortages for a majority of the drugs in the most dispensed IGs. Specifically, of the shortages considered to be most impactful, we were able to correctly predict 59% of them, one month in advance, without access to any inventory data from drug manufacturers and suppliers. This was achieved taking into account previous shortages, changes inherent in the constituent DINs, and the hierarchy of DINs within different IGs and therapeutic classes.

Even if the model cannot predict exactly what element of the supply chain will cause the shortage, the model remains useful to inform pharmacists’ drug ordering decisions. If a shortage is predicted for a particular drug in high demand, a pharmacist may decide to order more of that drug to serve his/her most vulnerable patients, while accepting the risk of having to mitigate the shortage by finding alternative drugs for other patients.

### Way ahead

While our forecasts had high accuracy for most IGs studied over the period studied, our models were weakest in quantifying the strength of the shortages, especially for the largest shortages. Further study taking into account other features (e.g., manufacturer reliability, shortages seen in or reported by other nations, and taking current supplier inventories into account) could possibly lead to more accurate forecasts.

We are now exploring different ways of putting the model into production as part of a suite of software already used by the Canadian pharmacies that generated the data used for this work. In particular, the way to present and explain forecasts as part of the software suite will be key to ensure results are well interpreted by pharmacists and useful to them.

Integrating shortage forecasts to the ordering and inventory management systems would be logical. Beyond the risk of shortage, which pertains to the supply-side of the pharmacy environment, we are currently working at better predicting the demand for drugs at each pharmacy using machine learning [48]. This forecasted demand, combined with the risk of shortage for certain drugs and other factors (e.g., inventory levels, risk tolerance) will be key in informing the drug ordering process. Once shortage predictions are integrated into the ordering process, it will be useful to do a before/after comparison, or A/B tests between pharmacies having access (or not) to the predictions, with a view to measuring how beneficial the model actually is.

## Declarations

• Mr. Mowaswes is the owner and CEO of PharmaGuide, the company that provided pharmacy data used in this work and plans to implement drug shortage predictions in PharmaGuide software.
